# Improving the efficiency of reactive case detection for malaria elimination in southern Zambia: a cross-sectional study

**DOI:** 10.1186/s12936-020-03245-1

**Published:** 2020-05-07

**Authors:** Fiona R. P. Bhondoekhan, Kelly M. Searle, Harry Hamapumbu, Mukuma Lubinda, Japhet Matoba, Michael Musonda, Ben Katowa, Timothy M. Shields, Tamaki Kobayashi, Douglas E. Norris, Frank C. Curriero, Jennifer C. Stevenson, Philip E. Thuma, William J. Moss, for the Southern and Central Africa International Centers of Excellence for Malaria Research

**Affiliations:** 1 MACS/WIHS Combined Cohort Study, Department of Epidemiology, Bloomberg School of Public Health, Johns Hopkins University, Baltimore, MD USA; 2 Division of Epidemiology and Community Health, School of Public Health, University of Minnesota, Minneapolis, USA; 3 Macha Research Trust, Choma District, Zambia; 4 Department of Molecular Microbiology and Immunology, Bloomberg School of Public Health, Johns Hopkins University, Baltimore, MD USA

**Keywords:** Malaria, Zambia, Elimination, Screening, Reactive case detection, Environment

## Abstract

**Background:**

Reactive case detection (RCD) seeks to enhance malaria surveillance and control by identifying and treating parasitaemic individuals residing near index cases. In Zambia, this strategy starts with passive detection of symptomatic incident malaria cases at local health facilities or by community health workers, with subsequent home visits to screen-and-treat residents in the index case and neighbouring (secondary) households within a 140-m radius using rapid diagnostic tests (RDTs). However, a small circular radius may not be the most efficient strategy to identify parasitaemic individuals in low-endemic areas with hotspots of malaria transmission. To evaluate if RCD efficiency could be improved by increasing the probability of identifying parasitaemic residents, environmental risk factors and a larger screening radius (250 m) were assessed in a region of low malaria endemicity.

**Methods:**

Between January 12, 2015 and July 26, 2017, 4170 individuals residing in 158 index and 531 secondary households were enrolled and completed a baseline questionnaire in the catchment area of Macha Hospital in Choma District, Southern Province, Zambia. *Plasmodium falciparum* prevalence was measured using *Pf*HRP2 RDTs and quantitative PCR (qPCR). A Quickbird™ high-resolution satellite image of the catchment area was used to create environmental risk factors in ArcGIS, and generalized estimating equations were used to evaluate associations between risk factors and secondary households with parasitaemic individuals.

**Results:**

The parasite prevalence in secondary (non-index case) households was 0.7% by RDT and 1.8% by qPCR. Overall, 8.5% (n = 45) of secondary households had at least one resident with parasitaemia by qPCR or RDT. The risk of a secondary household having a parasitaemic resident was significantly increased in proximity to higher order streams and marginally with increasing distance from index households. The adjusted OR for proximity to third- and fifth-order streams were 2.97 (95% CI 1.04–8.42) and 2.30 (95% CI 1.04–5.09), respectively, and that for distance to index households for each 50 m was 1.24 (95% CI 0.98–1.58).

**Conclusion:**

Applying proximity to streams as a screening tool, 16% (n = 3) more malaria-positive secondary households were identified compared to using a 140-m circular screening radius. This analysis highlights the potential use of environmental risk factors as a screening strategy to increase RCD efficiency.

## Background

In countries where a state of low-endemicity for malaria has been established and maintained, strategies and policies geared toward elimination of both vector and parasites have begun to take form. An essential component that has been incorporated in many of these elimination strategies is malaria control using environmental risk factors [1]. These strategies take advantage of the spatial distribution of malaria, which varies depending on the ecology and population, but in regions with low endemicity is often concentrated in small, isolated areas or “hot spots” comprised mostly of asymptomatic individuals still infectious to mosquitoes [2, 3]. Many malaria endemic countries have a surveillance system in place for identifying symptomatic cases in real-time (passive case detection or PCD); however, this system fails to reach asymptomatic individuals [1]. Active case detection (ACD) is a surveillance method recommended by the World Health Organization (WHO) in low transmission settings in which symptomatic and asymptomatic individuals are screened and treated for malaria [4]. Reactive case detection (RCD) is a form of ACD that was designed to take advantage of the spatial and temporal clustering of asymptomatic individuals within “hot spots” by using passively detected cases as triggers to initiate screening and treatment of individuals living in proximity to those cases [5, 6]. RCD is implemented in many countries working towards malaria elimination, including Zambia [6], South Africa [7], Brazil [8], Cambodia [9], and India [10].

The application of RCD in many of these settings differs in important features, such as the optimal screening radius and the number of households investigated [10]. In each instance, however, RCD is labour and time-intensive, requiring significant human resources, many rapid diagnostic tests (RDTs), and often long travel times between households [10, 11]. The utility of RCD in low transmission settings has been debated in part due to operational challenges during implementation and the use of less sensitive diagnostic tools such as RDTs and microscopy, which miss low density infections [1, 2, 10, 12]. Other limitations of RCD are its inability to reach populations with poor access, as well as the reliance on incident symptomatic cases seeking care to find “hot spots” comprised of asymptomatic individuals [10, 12].

Residual transmission in “hot spots” is driven by many local environmental factors such as vegetation and availability of aquatic habitats that determine vector density and heterogeneity [13]. For example, *Anopheles* larval sites contract and cluster around permanent aquatic habitats during the dry season, and expand during the wet season [14]. Various topographical features can also predict incident cases [13]. To increase efforts towards elimination, RCD may be improved by including environmental risk factors into the screening process, leveraging the heterogeneous nature of malaria transmission as a function of environmental features to guide asymptomatic case detection [13].

The Government of Zambia launched their RCD strategy in 2011 to enhance malaria surveillance and engage health systems at the community level to identify and treat individuals infected with *Plasmodium falciparum* who did not seek care or had minimal or no symptoms [15–17]. This RCD strategy is part of the National Malaria Elimination Strategic Plan (NMESP) to eliminate malaria in Zambia by 2021 and is employed in communities where parasite prevalence is approximately 1% and ten or fewer cases are passively detected at health facilities [15, 16, 18]. In Zambia, RCD starts with passive detection of a symptomatic malaria index case using *P. falciparum* histidine-rich protein-2 (*Pf*HRP2) RDTs at a rural health clinic or by community health workers (CHWs) at rural health posts. CHWs then perform household visits to screen-and-treat residents within the index household as well as neighbouring or secondary households within a 140-m radius [15].

Studies have shown that environmental risk factors can be used to identify households likely to have parasitaemic residents [19–24]; however, the use of such environmental risk factors have not been explored in southern Zambia in the context of RCD. Building on prior work that assessed the efficiency of RCD in southern Zambia, the predictive ability of environmental risk factors was evaluated at varied spatial scales to identify parasitaemic residents of households located within a larger radius of 250 m from an index household [18, 25, 26].

## Methods

### Study site

Households were enrolled into the RCD study in the catchment area of Macha Hospital in Choma District, Southern Province, Zambia between January 12, 2015 and July 26, 2017 [18, 25, 27, 28]. The region has a tropical savannah climate with the rainy season occurring from December to April, followed by a cool dry season from May to August, and a hot dry season from September to November as previously described [15, 18, 22, 26]. Malaria transmission is propagated by the primary vector *Anopheles arabiensis*, which peaks during the rainy season. Infections are almost exclusively due to *P. falciparum* [18, 25, 29]. The major malaria control interventions are case management with artemisinin-based combination therapy (ACT) introduced in 2004, long-lasting insecticide-treated nets (LLINs) that were introduced in 2007 and redistributed approximately every 3 years with the most recent being in November 2017, and targeted mass drug administration (MDA) and indoor residual spraying (IRS) largely outside the study area [18, 26].

### Reactive case detection

RCD eligibility and enrollment started at thirteen health centres and 23 health posts within the study catchment area where symptomatic individuals positive for malaria by *Pf*HRP2-based RDT (index cases) triggered follow-up visits by a CHW and study team from Macha Research Trust [15, 18]. The study field team received notifications of an index case through SMS text messages from the health centre staff, after which they visited the household of the index case as well as secondary households located within a 250 m radius of the index case within 1 week of notification [15]. The RCD radius was expanded from the government recommended 140 to 250 m for the study. If the index case travelled outside their home district in the previous month and stayed overnight, they were not eligible for RCD screening through the government program. The field team was trained to administer consent, perform RDT testing, provide ACT for uncomplicated malaria, collect finger prick blood on filter paper, administer surveys, collect data using electronic tablets, and educate participants on malaria transmission and prevention [18].

### Study population

The study population consisted of residents in an index case household and secondary households within 250 m of an index case. Households were single or multiple houses belonging to the main and extended family [30]. When index and secondary households were screened, all members of a household were eligible for enrollment. After written informed consent, including parental permission and assent for older children, a questionnaire was administered to obtain demographic information, knowledge of malaria transmission, malaria symptoms, travel history, and malaria prevention methods [18]. Parents or guardians completed surveys on behalf of participants younger than 16 years. A blood sample was collected for a *Pf*HRP2-based RDT (SD Bioline, Standard Diagnostics Inc, Gyeonggi-do, Republic of Korea) and as dried blood spots (DBS) on Whatman 903™ Protein Saver cards (GE Healthcare Bio-Sciences Corporation, Piscataway, NJ) for quantitative PCR (qPCR) [18, 19]. Household residents found to be RDT positive were offered artemether/lumefantrine (Coartem^®^), the standard treatment for uncomplicated malaria in Zambia. Global positioning system (GPS) coordinates were obtained at each household using hand-held GPSMAP^®^ 62 devices (Garmin Ltd, Olathe, Kansas) and mapped using ArcGIS version 10.5 (Environmental Systems Research Institute, Redlands, California) on a high resolution Quickbird™ satellite image of the catchment area [25, 28].

### Parasite prevalence

Parasite prevalence was determined using the *Pf*HRP2-based RDT results and detection of *P. falciparum* mitochondrial cytochrome b gene (*cytb*) by qPCR. *Pf*HRP2 RDT readings were performed according to the manufacturer’s instructions [31]. DBS samples for qPCR were stored in plastic bags with desiccants and transported to the laboratory at Macha Research Trust for further drying. Samples were re-packed and stored at − 20 °C until parasite DNA extraction was performed using the Chelex^®^ method [28, 32]. qPCR was performed with the Applied Biosystems StepOnePlus™ Real-Time PCR System (Thermo Scientific, Waltham, MA, USA). Primers specific to *P. falciparum cytb* were used to amplify, detect and quantify *P. falciparum* DNA using SYBR^®^ Green fluorescence [30, 33, 34]. Filter paper spotted with laboratory-cultured parasites (NF54) and dilutions of 3D7 genomic DNA were used as standards [32]. The limit of detection was established as one parasite/uL [18]. The qPCR reaction consisted of 5 µL DNA template, 5 µL SYBR^®^ Green PCR Master Mix (ThermoFisher), 200 nM forward primer (5′ CCT GAT AAT GCT ATC GTA 3′), and 200 nM reverse primer (5′ TAA TAC AAT TAC TAA ACC AGC 3′) [18]. Amplification with correct melting temperature was considered positive and the amplicon was further confirmed on a 4% agarose gel [18].

### Environmental risk factors

A Quickbird™ satellite image of the 1200 km^2^ catchment area provided by the GeoEye-1 satellite (DigitalGlobe Services, Inc., Denver, Colorado) in 2017 and comprised of four-band 1.64-m spatial resolution and 0.41-m resolution panchromatic single-band imagery. Remote sensing data was imported into ArcGIS version 10.5 to geocode index and secondary households [25, 28]. All data layers were projected onto the Universal Transverse Mercator (UTM), Southern Hemisphere, Zone 35, WGS1984. A digital elevation model (DEM) with 90-m resolution was obtained from Shuttle Radar Topography Mission (SRTM) version 3, processed in ERDAS Imagine 2011 software (Hexagon Geospatial, Madison, Al) and imported into ArcGIS [18, 26]. The ArcHydro Tools module of ArcGIS was used to build a stream network according to the Strahler stream classification that assigned order values of 1, 2, 3, etc. based on a hierarchy of tributaries, such that two small first-order streams join to form a second-order stream [19, 24, 35]. A shapefile for roads was created by digitizing roads in ArcGIS based on a 1:50,000 topographic map of Zambia and the satellite image. Households with one or more malaria positive individuals by RDT or qPCR (excluding the index case) were classified as positive households [19, 25]. Index and secondary households were compared based on the following baseline characteristics: median age of residents per household, number of individuals per household, number of individuals 5 years and younger per household, number of parasitaemic individuals, insecticide-treated bed net ownership, floor material, and cooking energy source [21].

Secondary households with and without parasitaemic individuals were also compared using the same variables. In addition to household descriptive variables, environmental risk factors characterizing the surroundings of secondary households and previously shown to be associated with malaria risk were evaluated on the following levels: (1) household-level, defined as environmental risk factors within 100-m radius of a household; (2) cluster-level, defined as environmental risk factors within 250-m radius of a household; and (3) neighbourhood-level, defined as environmental risk factors outside the 250-m screening radius.

**Fig. 1:**
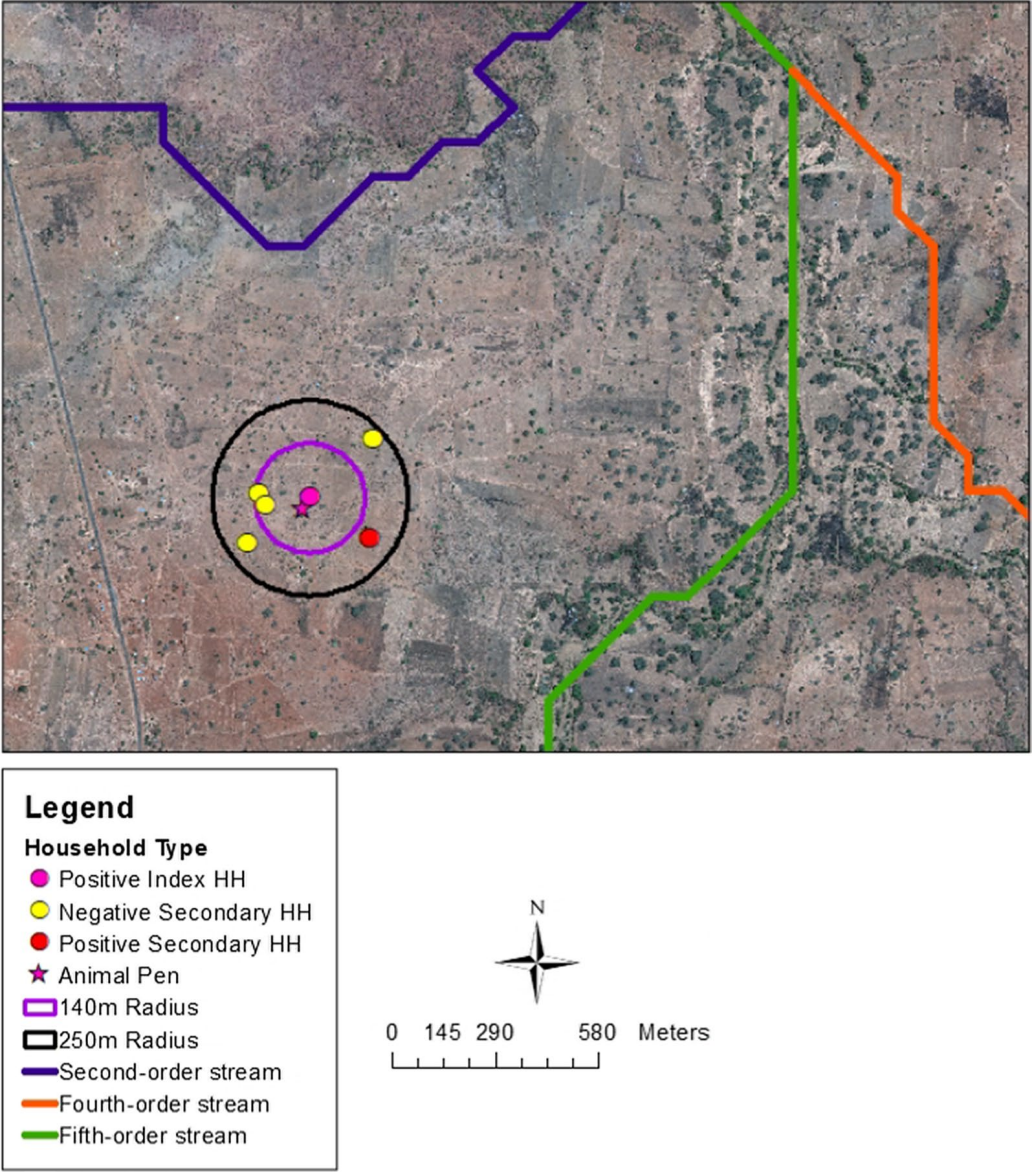
High-resolution Quickbird™ satellite image for catchment area of Macha Hospital in Choma District

Household-level risk factors included the number of animal pens within a 100-m radius of the main house structure and distance to nearest animal pens [36, 37]. Cluster-level risk factors included distance to index households and elevation difference with index households [20, 23, 24]. Neighbourhood-level risk factors included distance to the main road and distance to streams [19, 23]. If the distance between the index and secondary households was more than 300 m, coordinates were manually cross-referenced and re-mapped by the field team when necessary. Elevation differences were generated by taking the difference in elevation as recorded by GPS devices from each secondary household and its corresponding index household. Missing elevation coordinates were extracted from the DEM. Animal pens were manually digitized in ArcGIS and were defined as enclosed, dark- or light-brown, oblong, circular, or rectangular roofless structures of any size within a 100-m radius of the main house structure. Animal pens that were visually problematic to identify in ArcGIS were cross-referenced with Google Earth Images captured in 2017. From the individual stream order distances, the closest stream to the secondary household was identified. All risk factors generated using ArcGIS were imported into STATA 14.2 for statistical analyses. Figure 1 illustrates secondary households within the 140- and 250-m screening radius of the positive index household and the proximity of animal pens and streams.

### Statistical analysis

The Chi square test for proportions and Wilcoxon rank-sum for means were used to compare household descriptive variables between index and secondary households, as well as secondary households with and without parasitaemic individuals. The analysis was restricted to all participants in secondary households who provided consent, completed the survey, and had RDT or PCR results. Generalized linear models with inference based on generalized estimating equations (GEE) were used to estimate the cross-sectional population average effect for the difference in odds of a positive vs. negative secondary households for each environmental risk factor. The GEE model was chosen for its ability to account for the clustering of secondary households around the index household and to more accurately estimate standard errors. The outcome was a binary variable, distinguishing secondary households with parasitaemic individuals and those without. The household-, cluster-, and neighbourhood-level environmental risk factors were assessed for collinearity using variance inflation factor values. Variables included in the model were: distance to index household (per 50 m), distance to main road (per 50 m), elevation difference with index household (per 10 m), number of animal pens within 100 m, presence of animal pen (yes vs. no), season, and a categorical variable identifying nearest streams order 1 through 5. Model fit was evaluated using the Hosmer–Lemeshow goodness of fit test and significance was evaluated at a *p* value of 0.05 (Additional file 1: Table S1).

## Results

### Characteristics of the study population

Between January 12, 2015 and July 26, 2017, 4170 individuals (excluding index cases) in 689 households received a screening visit, completed a survey, and had RDT or qPCR result available. Of the 689 households, 77% (531) were classified as secondary households comprised of 2926 residents. The median number of individuals per households was 5 (interquartile range [IQR]: 3–7) and 7 (IQR: 5–10) (*P* < 0.001) for secondary and index households, respectively, with index households having more children 5 years and younger (median: 2.0, IQR 1.0–2.0) (*P* < 0.001). Almost half (46.8%) of index households had at least one parasitaemic individual other than the index case by qPCR or RDT, while only 8.5% of secondary households had at least one parasitaemic individual (*P* < 0.001). There were no differences in median age per household, insecticide-treated bed net ownership, household floor material, and preferred cooking energy between index and secondary households (Table 1).

**Table 1:** Household characteristics comparing index and secondary households enrolled from January 2015–July 2017

Household type	Secondary	Index	p-value^†^
N	531	158	
Median age per household, median (IQR)	17.6 (13.7, 25.1)	17.8 (14.8, 21.7)	0.66
Individuals per household, median (IQR)	5.0 (3.0, 7.0)	7.0 (5.0, 10.0)	< 0.001
Individuals 5 years and younger per household, median (IQR)	1.0 (0.0, 2.0)	2.0 (1.0, 2.0)	< 0.001
Number of parasitaemic individuals (RDT or qPCR)			< 0.001
0	486 (91.5%)	84 (53.2%)	
1	35 (6.6%)	56 (35.4%)	
2	9 (1.7%)	13 (8.2%)	
3	1 (0.2%)	5 (3.2%)	
Insecticide-treated bed net ownership			0.56
No bed nets	88 (16.8%)	21 (13.3%)	
One or more bed nets	429 (81.7%)	135 (85.4%)	
Do not know	8 (1.5%)	2 (1.3%)	
Household floor material			0.92
Natural (earth, mud, dung)	390 (74.4%)	120 (75.9%)	
Rudimentary (wood, planks)	3 (0.6%)	1 (0.6%)	
Finished flooring (parquet, tiles, brick, ceramic, concrete, carpet)	131 (25.0%)	37 (23.4%)	
Cooking energy source			0.86
Coal/charcoal	210 (40.0%)	62 (39.2%)	
Wood	315 (60.0%)	96 (60.8%)	

^†^Chi square test for proportions and Wilcoxon rank-sum for means

The median age within negative secondary households was 17.8 years (IQR: 13.7–25.4 years) and that within positive secondary households was 15.9 years (IQR: 13.1–20.7 years) (*P* = 0.16) (Table 2). The median number of individuals per households was 5 (IQR: 3–7) and 7 (IQR: 5–8) (*P* < 0.001) for negative and positive secondary households, respectively, with more individuals 5 years and younger residing in positive secondary households (*P *< 0.01) (Table 2). The composition of parasitaemic individuals in secondary households ranged from one (78% of households) to three individuals (2% of households) (*P *< 0.001). No differences were found in insecticide-treated bed net ownership, household floor material, and preferred cooking energy between positive and negative secondary households.

**Table 2:** Household characteristics comparing negative and positive secondary households enrolled January 2015–July 2017

Secondary household type	Negative	Positive	p-value^†^
N	486	45	
Average age per household, median (IQR)	17.8 (13.7, 25.4)	15.9 (13.1, 20.7)	0.16
Individuals per household, median (IQR)	5.0 (3.0, 7.0)	7.0 (5.0, 8.0)	< 0.001
Individuals 5 years and younger per household, median (IQR)	1.0 (0.0, 2.0)	2.0 (1.0, 3.0)	0.003
Number of parasitaemic individuals (RDT & qPCR)			< 0.001
0	486 (100.0%)	0 (0.0%)	
1	0 (0.0%)	35 (77.8%)	
2	0 (0.0%)	9 (20.0%)	
3	0 (0.0%)	1 (2.2%)	
Insecticide-treated bed net ownership			0.42
No bed nets	78 (16.3%)	10 (22.2%)	
One or more bed nets	394 (82.1%)	35 (77.8%)	
Do not know	8 (1.7%)	0 (0.0%)	
Household floor material			0.78
Natural (earth, mud, dung)	355 (74.1%)	35 (77.8%)	
Rudimentary (wood, planks)	3 (0.6%)	0 (0.0%)	
Finished flooring (parquet, tiles, brick, ceramic, concrete, carpet)	121 (25.3%)	10 (22.2%)	
Cooking energy source			0.34
Coal/charcoal	195 (40.6%)	15 (33.3%)	
Wood	285 (59.4%)	30 (66.7%)	

^†^Chi square test for proportions and Wilcoxon rank-sum for means

### Malaria prevalence

**Fig. 2:**
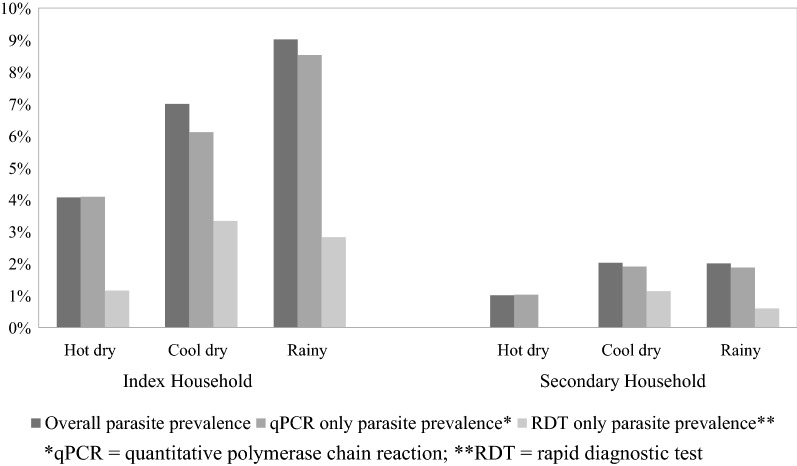
Parasite prevalence (%) for index and secondary households, January 2015–July 2017 in southern Zambia

Excluding index cases, 153 participants were positive for malaria by RDT or qPCR, 37% (56) of whom resided in 45 secondary households. The parasite prevalence for residents in secondary households was 0.7% using RDT and 1.8% using qPCR, while that for residents in index households was 2.7% and 7.3%, respectively. In secondary households, the parasite prevalence remained below 2.0% during the rainy, cool dry, and hot dry season, while a pattern of seasonal transmission was observed among index households with RDT parasite prevalence of 2.8%, 3.3%, and 1.2% during the rainy, cool dry, and hot dry season, respectively, and qPCR parasite prevalence of 8.5%, 6.1%, and 4.1%, for the corresponding seasons for index households (Fig. 2).

### Environmental household risk factors

Secondary households with parasitaemic individuals were not significantly further away from index households (median: 179.8 m, IQR: 121.7–226.9 m) compared to those without parasitaemic individuals (median: 164.7 m, IQR: 104.1–210.1 m) (*P* = 0.17) but they were in closer proximity to streams (median: 335.3 m, IQR: 242.2–539.1 m) (*P* < 0.01) (Table 3). Although no statistically significant differences were detected for the other environmental risk factors, positive secondary households exhibited a trend previously associated with increased malaria risk [19–21, 26, 36–38]: they were at lower elevation from the index household (median: − 0.7, IQR: − 8.9 to 9.4 m) (*P* = 0.52); further away from the main road (median: 9266.6 m, IQR: 4130.4–17,353.7 m) (*P* = 0.28); and more likely to have an animal pen (64.4%) (*P* = 0.69). If an animal pen was present, they were also closer to it than negative secondary households (median: 36.9 m, IQR: 20.6–50.5 m) (*P* = 0.43) (Table 3).

**Table 3:** Environmental household risk factors comparing negative and positive secondary households enrolled January 2015–July 2017

Secondary household type	Negative	Positive	p-value^†^
N	485^a^	45	
ESD radius			0.56
≤ 140 m	194 (40.0%)	16 (35.6%)	
140–250 m	291 (60.0%)	29 (64.4%)	
Distance to index household in metres, median (IQR)	164.7 (104.1, 210.1)	179.8 (121.7, 226.9)	0.17
Elevation difference with index household in metres, median (IQR)	0.0 (− 9.3, 11.0)	− 0.7 (− 8.9, 9.4)	0.52
Distance first-order stream in metres, median (IQR)	691.0 (339.6, 1033.0)	485.9 (307.5, 833.1)	0.081
Distance second-order stream in metres, median (IQR)	2008.8 (1029.5, 3498.0)	1578.8 (1029.3, 2873.5)	0.21
Distance third-order stream in metres, median (IQR)	3368.5 (1541.0, 5950.5)	3391.8 (1494.9, 5350.4)	0.63
Distance fourth-order stream in metres, median (IQR)	5363.7 (2233.4, 9001.8)	6043.9 (2503.6, 9395.2)	0.63
Distance fifth-order stream in metres, median (IQR)	5291.9 (1651.4, 8297.5)	5382.0 (1405.2, 10061.5)	0.95
Distance sixth-order stream in metres, median (IQR)	35003.7 (30907.1, 41962.9)	34138.1 (27043.0, 41075.6)	0.59
Distance Nearest Stream in metres, median (IQR)	533.2 (275.1, 795.5)	335.3 (242.2, 539.1)	0.006
Nearest stream order in metres			0.21
First	322 (66.4%)	25 (55.6%)	
Second	50 (10.3%)	3 (6.7%)	
Third	37 (7.6%)	7 (15.6%)	
Fourth	21 (4.3%)	2 (4.4%)	
Fifth	55 (11.3%)	8 (17.8%)	
Distance main road in metres, median (IQR)	8457.1 (3121.1, 13666.0)	9266.6 (4130.4, 17353.7)	0.28
Distance nearest animal pen in metres, median (IQR)	38.6 (25.7, 59.4)	36.9 (20.6, 50.5)	0.43
Number of animal pens within 100 m, median (IQR)	1.0 (0.0, 2.0)	1.0 (0.0, 1.0)	0.92
Animal pen			0.69
No	187 (38.6%)	16 (35.6%)	
Yes	298 (61.4%)	29 (64.4%)	

^†^Chi square test for proportions and Wilcoxon rank-sum for means

^a^One household outside 250-m radius of index household excluded

### Efficiency of reactive case detection

The association between positive secondary households and environmental risk factors at the household-, cluster-, and neighbourhood-level, was evaluated for 45 positive and 485 negative secondary households. One negative secondary household was excluded as it was > 300 m from the index household. Cluster- and neighbourhood-level risk factors were associated with positive secondary households in the multivariate GEE model, while no significant association was observed for household-level risk factors (Table 4). The cluster-level risk factor that was marginally associated with a positive secondary household was increasing distance to the index household (*P *= 0.07). As the distance to index households increased by 50 m, the odds of a positive secondary household increased by 24% (adjusted odds ratio [OR]: 1.24, 95% confidence interval [CI] 0.98–1.58). No association was observed for elevation. The neighbourhood-level risk factor associated with positive secondary households were third- and fifth-order streams. Positive secondary households were 3.14 times more likely to be located near a third-order stream (OR: 2.97, 95% CI 1.04–8.42) (*P *= 0.041) and 3.20 times more likely to be located near a fifth-order stream (OR: 2.30, 95% CI 1.04–5.09) (*P *= 0.040), compared to a negative secondary household. Fourth-order streams (OR: 1.62, 95% CI 0.21–12.65) also exhibited a trend of increased risk; however, the association was not statistically significant (*P *= 0.64). No association was observed for the distance to the main road. The household-level risk factor of number of animal pens within 100-m was not associated with positive secondary households; however, if an animal pen was present, the odds of being a positive household increased by 60% (OR: 1.60, 95% CI 0.57–4.47), although the association was not statistically significant (*P *= 0.37).

**Table 4:** Crude and adjusted OR for the association between environmental risk factors and positive secondary households

Risk Factors	Crude OR	Adjusted OR
Distance to index household (per 50 m)	1.21 (0.95, 1.54)	1.24^ϯ^ (0.98, 1.58)
Distance to main road (per 50 m)	1.00 (0.99, 1.00)	1.00 (1.00, 1.01)
Elevation difference with index household (per 10 m)	1.00 (0.97, 1.03)	1.00 (0.94, 1.08)
Number of animal pens	1.01 (0.75, 1.37)	0.95 (0.60, 1.48)
Animal pen present		
No	Ref	Ref
Yes	1.14 (0.55, 2.34)	1.60 (0.57, 4.47)
Nearest stream order		
First	Ref	Ref
Second	0.77 (0.23, 2.65)	0.66 (0.17, 2.48)
Third	2.44^¥^ (0.98, 6.08)	2.97^*^ (1.04, 8.42)
Fourth	1.23 (0.16, 9.56)	1.62 (0.21, 12.65)
Fifth	1.87 (0.81, 4.32)	2.30^*^ (1.04, 5.09)
Season		
Cool dry season (May–Aug)	Ref	Ref
Hot dry season (Sep–Nov)	0.38 (0.08, 1.87)	0.49 (0.10, 2.47)
Rainy season (Dec–Apr)	1.05 (0.52, 2.13)	1.37 (0.66, 2.83)

Exponentiated coefficients; 95% confidence intervals in brackets

^†^p-value for adjusted OR for distance to index household is marginal (p = 0.074)

^**¥**^ p-value for crude OR for nearest stream category 3 is marginal (p = 0.056)

^*^p < 0.05

An increased risk of identifying a positive secondary household near streams suggests that environmental features could potentially guide RCD screening strategies. To evaluate if this increased probability of identifying secondary household with parasitaemic individuals would require fewer households to be screened, streams closest to an index household were used to find secondary households within the 250-m radius. Using only streams, a total of 137 secondary households, 14% (n = 19) that had parasitaemic residents, representing 42% of all positive secondary households in the study sample, would have been identified. The current RCD screening method using only the 140-m radius identified 210 secondary households; however, only 8% (n = 16) of these secondary households had parasitaemic residents, representing 36% of all positive secondary households in the study sample. These results indicate that incorporating environmental risk factors such as streams, within a larger screening radius, would allow for more parasitaemic individuals to be identified while screening fewer number of households, consequently increasing the efficiency of the RCD programme [25]. If streams nearest to index households were used to find secondary households within a 250-m radius, 16% (n = 3) more secondary households with parasitaemic residents would have been screened and treated compared to the national RCD strategy with 140-m screening radius.

## Discussion

Environmental risk factors were associated with the probability of finding households with parasitaemic residents using RCD as demonstrated in other studies in Zambia [13]. In the low transmission setting of Choma District, Zambia, identifying streams located near index households to guide and direct screening has the potential to improve RCD and affect transmission by identifying households with asymptomatic infections. These findings are in line with a previous study conducted within the same study area in 2008 where it was shown that households within 1.98 km from a third-order stream were 2.8 (95% CI 1.2–6.9) times more likely to have an RDT positive resident than those within 6 km [26].

Although no associations were found with the other environmental risk factors such as distance to a main road, elevation, season, and number and presence of animal pens, non-parametric comparisons between positive and negative secondary households exhibited a trend of increased malaria risk for these risk factors [19–21, 26, 36–38]. The risk associated with animal pens varies in the literature depending on vector behaviour. *Anopheles arabiensis* has been reported to be anthropophilic in southern Zambia but also displays zoophilic habits by feeding opportunistically on non-human blood sources [36]. Other vectors besides *An. arabiensis* might also have an important role in transmission as *P. falciparum*-infected *Anopheles squamosus* exhibiting outdoor zoophagic feeding behaviour were recently identified in the area. Early studies in Choma District, Zambia found that ownership of cattle reduced the risk of *P. falciparum* infection by 87% while others have found less conclusive evidence [36, 37]. For elevation, however, it has been clearly shown that increased elevation offers protection against malaria infection [13, 20, 24, 26, 38]. Since index and secondary households in this study were located only < 300 m from each other and variation in elevation was minimal, it is unlikely that the elevation would influence malaria risk at this spatial scale. Distance from the index household marginally increased the probability of finding positive secondary households (OR: 1.24, 95% CI 0.98–1.58), in contrast to other studies. Larsen et al. observed a decreased risk for neighbouring households located further away, and Bulterys et al. found an adjusted OR of 0.26 (95% CI 0.07–0.98) as distance between households increased. Finally, distance to the main road has often been treated as an indicator of increased malaria risk. In Chongwe District, Zambia, the odds of RDT positive households increased by 5% for every 500-m increase in distance from the road [39]. As only proximity to the main road was examined, it is possible that constant use from vehicles, animal carts, and people prevented mosquito breeding sites from developing undisturbed, reducing this as a risk factor. Less frequently used subsidiary roads and rural paths (not included in the analysis) could provide more opportune mosquito breeding sites closer to residences as their composition allows for easier accumulation of aquatic breeding sites compared to the tarmac and concrete main road.

The use of environmental risk factors for malaria risk prediction is a common approach to malaria control and has been employed in various transmission settings around the world. For example, in Chimoio, Mozambique a GIS-based spatial model was designed to estimate areas of risk using temperature, precipitation, altitude, slope, distance to water bodies, distance to roads, normalized difference vegetation index (NDVI), land use, and land cover [40]. The model identified that 4% of Chimoio was at high risk for contracting malaria, with precipitation as a key risk factor for the entire area studied [40]. Another study in south Sumatra, Indonesia used ordinary least square and geographically weighted regression to show that altitude, distance to forest, and rainfall determined overall malaria incidence with considerable heterogeneity at the village level [41]. These findings were consistent with other studies in Cambodia, Addis Ababa, Ethiopia, and Rondôia, Brazil [41]. Despite the extensive literature on environmental risk factors for malaria, their application within the context of RCD has been limited.

Many studies evaluating the efficiency of RCD highlight its inability to halt infections in areas of low transmission due to the use of less sensitive RDTs, travel-related infections, and large budgetary requirements [2, 18]. A major concern for RCD-based strategies is that asymptomatic individuals will be missed if no clinical cases report to CHWs [42]. A survey in coastal Kenya found that asymptomatic and symptomatic infections do not necessarily overlap spatially, and that clusters of symptomatic infections have greater temporal stability over more than 10 years [42]. Another issue often highlighted is the different criteria and screening radii employed by countries to define and recruit neighbouring households [42] For example, RCD data from four villages in the Myanmar-Thailand border determined that RCD would only be successful at a radius of 150 m, and any screening occurring beyond this radius would not perform better than random screening [2]. Another study in Pailin Province in western Cambodia screened the nearest five households for every fifteenth index case and ten nearest households for every 30th index case. Using this approach, they predicted only 40% of infections and concluded that RCD was not recommended in a setting targeting elimination [43]. However, with the shortcomings of a circular radius and the various implementation challenges, for RCD to be an effective method for malaria elimination in these low-endemic countries a tailored approach adapted to the local parasite epidemiology, vector biology, and living/working environment of the community must be considered key for it to succeed.

This study used environmental risk factors for malaria to characterize the low transmission setting to improve RCD efficiency. Previous work on enhancing RCD efficiency in Southern Province, Zambia has also shown that time-invariant measures of the environment, such as the topographic position index (TPI; measure of an area’s relative elevation to find slopes, valleys, and ridges), the convergence index (CI; measure of an area’s propensity to pool water), median enhanced vegetation index (EVI; measure of vegetation density), and the topographical wetness index (TWI; measure of water flow) were stronger predictors for identifying parasitaemic individuals than demographics of incident symptomatic cases [13, 26]. They showed that ridges and upper slopes (at a TPI scale of 270 m) and wetter regions (TWI > 10.2) were associated with finding more parasitaemic individuals during RCD [13]. These findings, along with the current study, support the significance of water bodies in improving the efficiency of RCD. Third through fifth-order streams are mid-level streams that may not always be suitable for larval development; however, larvae have been collected from water at the edges of these streams (unpublished findings). During the dry season, as water accumulates into smaller pools, they become ideal larval development sites. These streams can also serve as important markers for nearby areas with similar high water table harbouring larvae [13, 26, 38, 44]. And as these streams can be challenging to locate depending on size and season, spatial risk maps with topographical measures, such as CI and TWI, can offer guidance to CHWs to possibly reach clusters of asymptomatic carriers otherwise missed during regular RCD screening. Other water sources such as dams, are also important determinants for malaria transmission as was shown in Ethiopia, where reservoir water level management suppressed larval development [45].

In addition to the use of streams as a screening tool, RCD efficiency could benefit from the combined use of RDTs and highly sensitive qPCR. For this study region, the overall parasite prevalence (3.7%) was mostly driven by qPCR as parasite prevalence by RDT was only 1.3%. Although costly, sensitive molecular methods such as qPCR are critical in low endemic settings to detect potential parasite-transmitting asymptomatic carriers. Even ultra-sensitive RDTs (uRDTs), such as the new Alere™ Malaria Ag P.f uRDT which was designed for low transmission settings, may not be sufficiently sensitive alternatives to SD Bioline *Pf*HRP2 RDTs [46]. The Alere™ us-RDT has a ten-fold lower limit of detection for PfHRP2 compared to regular RDTs but missed 56% of PCR-detectable *P. falciparum* infections in a low-endemic setting in Myanmar, and in Papua New Guinea the test missed 50% of *P. falciparum* infections otherwise detectable by qPCR [47].

There were several limitations to this study. Restricting environmental variables within set radii raises concerns for edge-effect associations. For example, animal pens located just outside the 100-m radius were not counted as belonging to neighbouring households, thus potentially underestimating the number of animal pens associated with a household. Not all environmental risk factors important for malaria transmission were evaluated. Vegetation cover, an important indicator of available mosquito habitat, could also be a useful screening tool and has yet to be evaluated for RCD strategies [13, 48, 49]. Finally, the risk factors shown to be associated with positive households in this low transmission setting of Choma District, Zambia may not be applicable in other endemic regions.

The effectiveness of RCD ultimately depends on the number of cases found and treated in a timely manner and the resources allocated during implementation [2]. However, it is important to consider the added value of a tailored RCD approach based on demographic and ecological risk factors and more sensitive diagnostic tools to fully reap the benefits of this screening method to achieve malaria elimination [1]. In Cambodia, where infection is linked to occupation and mobility, an expanded RCD approach was implemented in which individuals who were coworkers of a symptomatic index case in settings of high malaria infection, such as forests and plantations, were also screened [1]. The expanded RCD had a detection rate of 3.9% compared to 0–2% using the classic RCD approach [1]. Through this adapted RCD design, Cambodia’s National Malaria Control Programme sought to identify and treat asymptomatic individuals within a discrete population whose members shared a common malaria risk through occupations such as logging, mining, and migrant labour [1]. The environmental risk factors identified in this study demonstrate that, even in low transmission settings, a tailored approach is possible; however, further work is needed to fully understand how these risk factors vary across district and season and how they can be modified to guide RCD strategies nationally in Zambia.

## Conclusion

This study identified higher order streams as risk factors for parasitaemia in households neighbouring an index case as part of RCD in rural southern Zambia. These risk factors have the potential to improve the efficiency of RCD in a low transmission setting by not only identifying parasitaemic individuals more efficiently but also potentially reducing the number of households needed to be screened. Combined with other strategies for malaria elimination, tailored RCD approaches can help realize the goal of malaria elimination in Zambia.

## Supplementary information

**Additional file 1: Table S1.** QIC goodness-of-fit statistics for the GEE models.

## Data Availability

The datasets used and/or analysed during the current study are available from the corresponding author on reasonable request and through ClinEpiDB (https://clinepidb.org/ce/app/).

## Abbreviations

ACD: Active case detection
ACT: Artemisinin-based combination therapy
aOR: Adjusted odds ratio
CHW(s): Community health worker(s)
CI: Convergence index
*Cytb*: Cytochrome b gene
DBS: Dried blood spots
DEM: Digital elevation model
EVI: Enhanced vegetation index
GEE: Generalized estimating equations
GIS: Geographic information system
GPS: Global positioning system
IRS: Indoor residual spraying
IQR: Inter quartile range
LLINs: Long lasting insecticide treated nets
MDA: Mass drug administration
NDVI: Normalized difference vegetation index
NMESP: National Malaria Elimination Strategic Plan
OR: Odds ratio
PCD: Passive case detection
PfHRP2: *P. falciparum* histidine-rich protein-2
qPCR: Quantitative polymerase chain reaction
RCD: Reactive case detection
RDT(s): Rapid diagnostic test(s)
SRTM: Shuttle Radar Topography Mission
TPI: Topographic position index
TWI: Topographical wetness index
uRDT(s): Ultra-sensitive RDT(s)
UTM: Universal transverse mercator
WHO: World Health Organization
