# Supplementary material for: Improving the efficiency of reactive case detection for malaria elimination in southern Zambia: a cross-sectional study
Source: Malar J. 2020 May 7;19:175. doi: 10.1186/s12936-020-03245-1 (PMC7206707; doi:10.1186/s12936-020-03245-1)
Supplement: Supplementary file 1 — Additional file 1: Table S1. QIC goodness-of-fit statistics for the GEE models. [file 12936_2020_3245_MOESM1_ESM.docx]

**Table**

QIC goodness-of-fit statistics for the GEE models

| Correlation | Variable | P | Trace | QIC | QIC_u_ |
| --- | --- | --- | --- | --- | --- |
| Independent | All* | 8 | 7.94 | 314.09 | 314.22 |
| Independent | Excluding nearest stream order and season | 6 | 6.16 | 315.66 | 315.34 |
| Independent | Excluding distance to index household and distance to main road | 6 | 6.10 | 315.54 | 315.33 |
| Independent | Excluding distance to main road | 7 | 7.05 | 315.46 | 315.36 |
| Independent | Excluding nearest stream order | 7 | 7.27 | 317.65 | 317.11 |
|  | | | | | |
| Exchangeable | All* | 8 | 7.63 | 314.75 | 314.49 |
| *Distance to index household (per 50m), Distance to main road (per 50m), Elevation differenc with index household (per 10m), Number of animal pens, Animal pen present, Nearest stream order, Season. | | | | | |
